# Odor exploration behavior of the domestic pig (*Sus scrofa*) as indicator of enriching properties of odors

**DOI:** 10.3389/fnbeh.2023.1173298

**Published:** 2023-05-05

**Authors:** Maria Vilain Rørvang, Sarah-Lina Aagaard Schild, Johanna Stenfelt, Rebecca Grut, Moses A. Gadri, Anna Valros, Birte L. Nielsen, Anna Wallenbeck

**Affiliations:** ^1^Department of Biosystems and Technology, Swedish University of Agricultural Sciences, Lomma, Sweden; ^2^Innovation Centre for Organic Farming, Aarhus, Denmark; ^3^Department of Production Animal Medicine, Faculty of Veterinary Medicine, Research Centre for Animal Welfare, University of Helsinki, Helsinki, Finland; ^4^Universities Federation for Animal Welfare (UFAW), Wheathampstead, United Kingdom; ^5^Department of Animal Environment and Health, Swedish University of Agricultural Sciences, Skara, Sweden

**Keywords:** olfaction, smell, pig production, sensory enrichment, animal welfare, sniffing, agonistic behavior, pig behavior

## Abstract

**Introduction and aim:**

Although the sense of smell in pigs is widely recognized as being highly developed, surprisingly little is known about their sensory ability. This study aimed to (a) identify which non-social odors pigs were able to detect and distinguish between, (b) investigate the types of behavior expressed when exploring odors and, (c) compare pigs’ responses to the different odors to evaluate their interest in the odors.

**Methods:**

Growing pigs (*N* = 192) of crossbred commercial breeds were enrolled in the experiment (32–110 days of age, weighing 64.9 ± 10.1kg). Littermate pairs of opposite sex were tested in test pens with two odor insertion points in the pen wall, 55 cm apart. All pigs were habituated to the test pens and experimenters. Twelve odors were tested (eight essential oils and four synthetic perfumes) in groups of three odors, with each pig pair tested once with one set of three odors (all possible orders of the three odors were tested on 24 pairs in total), always against a non-odor control (demineralized water). In a test, each of the three odors were presented during three trials in a row (a total of 9 trials per test; trial duration: 1 min; inter-trial breaks: 2 min; total test duration: 25 min). Response variables included: duration of sniffing, feeding-related behavior (licking, biting and rooting), agonistic behavior (biting, displacement and pushing) and no approach of the odor or control, recorded throughout each 1-min odor presentation.

**Results:**

All pigs sniffed an odor less when repeatedly presented (LMM: all odors *P* < 0.05), and significantly longer at the subsequent presentation of a new odor [LMM (3^rd^ vs. 1^st^ presentations): *P* < 0.001]. Specific odor and odor type (essential oil vs. synthetic perfume) had no significant effect on sniffing duration. Overall, feeding-related behavior and agonistic behavior were expressed significantly more when pigs explored the odor compared with the control insertion point (Paired *t*-tests: *P* < 0.001), and specific odor only affected the expression of feeding-related behavior.

**Conclusion:**

Collectively, pigs express sniffing, agonistic, and feeding-related behavior when exploring odors, which suggests that pigs perceive odors of non-social origin as a resource. Odors may thus constitute relevant enrichment material for pigs.

## 1. Introduction

### 1.1. Olfaction of pigs

The domestic pig (*Sus scrofa*) is considered to have well-developed olfactory abilities. This is epitomized in the use of pigs to hunt for truffles, an expensive and valued delicacy that grows underground. The sense of olfaction develops early in the piglet’s life, and olfactory cues play an important role in social communication (Kittawornrat and Zimmerman, 2011). It is therefore surprising that the use of odors in the practical management of pigs in modern pork production has received little attention. Scientific studies on the aforementioned topic have focused mainly on aspects deemed relevant for optimizing the economy of the production: palatability of feed for pigs to increase feed intake (Jacela et al., 2010), odors when discriminating between conspecifics (Meese and Baldwin, 1975; Kristensen et al., 2001), odors (boar smell/androstenone) to stimulate sow estrus [e.g., (Booth and Signoret, 1992; Sorensen, 1996; Rekwot et al., 2001)], prenatal odor exposure (via the sow’s feed) and subsequent postnatal flavor recognition in piglets (Oostindjer et al., 2009), studies examining the role of the vomeronasal organ (Asproni et al., 2022; Mechin et al., 2022) and the processing of pheromones in pigs (McGlone, 1985; Guiraudie et al., 2003; Salazar et al., 2003, 2004; Dinka et al., 2016), and the role of the main olfactory system in detecting pheromones (Dorries et al., 1997). No previous studies investigating the broader olfactory capacities of the pig could be found and, to our knowledge, only one study has compared pigs’ interests in various odors. Nowicki et al. (2015) provided weaned piglets with odorized enrichment objects (i.e., toys) and found that piglets showed more interest in objects odorized with natural odors, spending more time near objects smelling of moist soil, grass or dried mushrooms than of synthetic odorants; vanilla, strawberry and orange. Although Nowicki’s study indicates a potential to incorporate such odors in environmental enrichment strategies for pigs, literature on pigs’ odor-exploration behavior and general perception of odors of non-social origin (i.e., odors not from conspecifics) is lacking.

### 1.2. Odors as enrichment for pigs

Pigs have an inherent motivation to explore their surroundings and will, when given the opportunity, spend a large part of their awake time foraging, licking, sniffing, and rooting (Horsted et al., 2012; Martinez-Macipe et al., 2020). It is well-known that barren environments and limited space, which are often seen in modern intensive pork production, have negative welfare implications for the pig (Kallio et al., 2018). Providing environmental complexity through, for instance, rooting material and/or enrichment materials or objects, creates more opportunities for the pigs to express explorative and appetitive behaviors, with positive effects on pig welfare in terms of behavior (Bolhuis et al., 2005; Van de Weerd et al., 2005; Van De Weerd et al., 2006; Telkanranta et al., 2014), cognition (de Jong et al., 2000; Sneddon et al., 2000; Douglas et al., 2012), and physiology (Beattie et al., 2000; Luo et al., 2020; Wen et al., 2021). A problematic aspect of environmental enrichment is the often rapid habituation to a provided stimulus (Tarou and Bashaw, 2007), thereby reducing its novelty and limiting its value and effectiveness. The effectiveness of environmental enrichment for pigs lies in its capacity to help the pig adapt to its environment, for instance by increasing the opportunities to perform species-specific behavior (van de Weerd and Day, 2009). Odors may be a way to enrich the pigs’ environment, either in themselves or by increasing and potentially prolonging pigs’ interest in enrichment materials or objects. From other mammalian species kept in environments with limited space and complexity, olfactory enrichment has proven to affect a variety of behaviors positively (e.g., activity, explorative, and social behavior) in zoos [primates: (Gronqvist et al., 2013; Wowk and Behie, 2023), felids: (Powell, 1995; Wells and Egli, 2004; Yu et al., 2009)], and in shelters [dogs: (Graham et al., 2005; Binks et al., 2018; Murtagh et al., 2020), cats: (Ellis and Wells, 2010; Machado and Genaro, 2014)]. It is therefore reasonable to hypothesize that olfactory enrichment holds the potential to positively impact other animals with a keen sense of smell kept in environments of similar constraints, including farm animals in intensive production.

In the Nowicki et al. (2015) study mentioned above, piglets maintained a higher interest in odorized compared with odorless objects over time, and periodic changes in odors increased and prolonged the attractiveness of the objects. Such findings suggest that novelty is a key factor in pigs’ motivation for odor exploration. The value of an environmental enrichment is linked to the animal’s motivational state (Arnott and Elwood, 2008) and cost-benefit for the animal (Brown, 1964) of the enrichment. Fighting for access to an enrichment is associated with high costs and the economic defendability of a resource, therefore, depends on the pigs’ perceived benefit of gaining access to it (Fraser et al., 1995). An enrichment therefore only has economic defendability when the value supersedes the cost (Brown, 1964), and the higher the perceived value, the higher the willingness to “pay” (make an effort) to gain access (Arnott and Elwood, 2008). Hence, looking at the social behavior of pigs when exposed to olfactory enrichment could give insight into what pigs assess the resource value of different odors to be.

In the 1980’s, the olfactory habituation/dishabituation test was developed as a method to assess whether animals can detect and distinguish between different odors (Sundberg et al., 1982). The test was first used to assess the olfactory capacities of gerbils (Gregg and Thiessen, 1981) but has since then been used for other species [cattle: (Rørvang et al., 2017), horses: (Hothersall et al., 2010; Rørvang et al., 2022), pigs: (Mendl et al., 2002)], including a recent study on the sensitivity of gilts to boar pheromone (Aviles-Rosa et al., 2020). The aim of the current study was to investigate the olfactory capacities of pigs using an adapted version of the habituation/dishabituation paradigm. Specifically, the study aimed to (a) identify which non-social odors pigs were able to detect and distinguish between, (b) investigate what types of behavior are expressed when exploring odors and, (c) use the observations from (a) and (b) to compare pigs’ responses to the different odors to evaluate their interest in the respective odors.

## 2. Materials and methods

### 2.1. Ethical considerations

The experiment was approved by the Swedish Board of Agriculture’s Uppsala Ethics Committee on Animal Research (ethics approval number Dnr. 5.2.18-02900/2020), in compliance with EC Directive 86/609/EEC on animal studies. All procedures were conducted in accordance with the ethical guidelines proposed by the Ethics Committee of the ISAE [The International Society of Applied Ethology; (Sherwin et al., 2003)] and met the ARRIVE guidelines (Kilkenny et al., 2010).

### 2.2. Animals and experimental conditions

**FIGURE 1:**
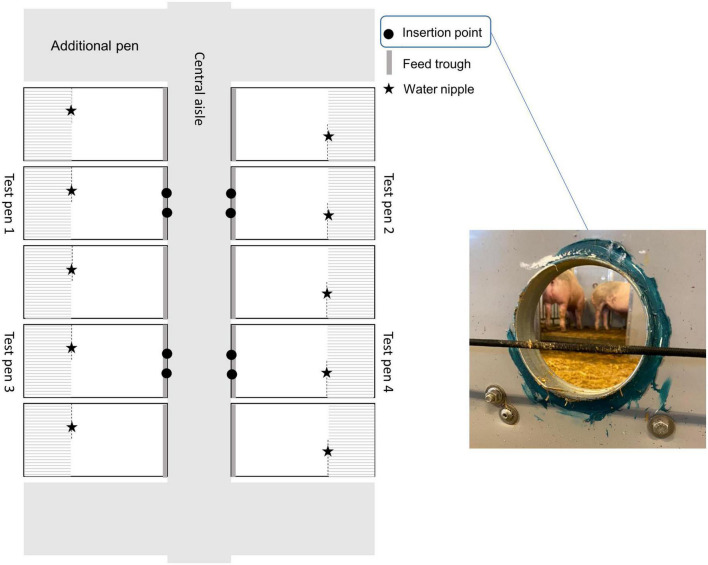
Top view of the experimental room within the experimental building with five pens on either side of the central aisle, each with a slatted and a solid floor area. The four test pens had, in addition to the usual pen equipment, two odor insertion points in the front wall of the pen. Placing of the water nipple and the feed trough is indicated. Littermate pairs occupied both test pens and non-test pens on both sides of the central aisle. Each * represents a water nipple.

The experiment was carried out from February to July 2022 at facilities for growing-finishing pigs at the Swedish Livestock Research Centre at SLU, Uppsala, Sweden. The building where the experiment was carried out was selected to minimize disturbances in the experiment from other experiments or daily activities at the center, and to comply with COVID-19 regulations. The air inside the building was electronically monitored and ventilated via the building’s ventilation system (vacuum system, Fancom, Panningen, Netherlands). Temperature in the building varied from an average daily temperature of 15^°^C in February to 21^°^C in June. Seven pens for growing pigs (length × width: 335 cm × 177 cm) were located on either side of a central aisle, i.e., a total of 14 pens distributed over two pen rows. Of these, five pens on each side were used in the experiment with the end pens of each row left empty. Each pen consisted of a concrete floor area [length (including feed trough) × width: 217 cm × 177 cm] and a slatted area (length × width: 118 cm × 177 cm) elevated 19 cm above the floor. From the concrete floor, pigs had access to a feed trough (length × width: 177 cm × 23 cm) with wet feed (Opti finish, Svenska foder, Lidköping, Sweden) provided three times daily at approx. 0600, 1,200 and 1,800 h. In the slatted area, pigs had access to an automatic drinker (water flow: 3 L/min). Pigs were given two large handfuls of chopped barley straw daily on the solid floor. Four of the pens, two on either side of the central aisle, were test pens (Figure 1, test pens 1–4), fitted with two odor insertion points in the front (aisle facing) wall of the pen, 55 cm apart (measured at the center of each point). The insertion points consisted of a hole (Ø: 14 cm) drilled through the pen wall and fortified with a metal ring to prevent pigs from harming their snout on any rough edges when exploring the hole. The diameter of the holes was chosen to allow sufficient space for the pigs to insert their snouts, without being able to make direct contact with the plastic box used for odor presentations (each odor box was 17 cm deep, see section “2.5 Preparation of odor samples” for more details). All insertion points were made approximately 1 month before testing, to allow potential odors from the newly exposed wall edge and the metal ring to dissipate.

Growing pigs (*n* = 192; 32–110 days of age, mean body weight ± SD: 64.9 ± 10.1 kg) of crossbred commercial Swedish breeds (Yorkshire x Hampshire *n* = 149, Duroc x Yorkshire *n* = 8, Landrace x Hampshire *n* = 35) originating from 52 birth litters, were included in the experiment. The range in ages was chosen to enable the study of potential age effects and was also the main cause of the relatively large weight range. The pigs were moved from their home pen [for details about standard management routines see: (The Swedish Livestock Research Centre, 2017)] to the experimental building in littermate groups of 8–10 pigs depending on litter size. Two groups were moved on the same day and upon arrival, all pigs were individually weighed and spray-marked for easy identification. Each group was then divided randomly into pairs of opposite sex (4–5 pairs per littermate group depending on group size and sex ratio) and housed in these pairs in the pens inside the experimental building. The moving, marking and pairing were done by the farm staff who was blind to the treatments. Each littermate group thus occupied one pen row of 4–5 adjacent pens with one littermate pair per pen (Figure 1). The pigs were acclimatized for 24–29 h to ensure that all pigs were familiar with the experimental environment and the insertion points. During this acclimatization period, the pigs were able to investigate the insertion points by sniffing, touching, licking, biting and pushing the holes. The holes were covered on the outside with a box but no odor was present during this period. This acclimatization process was repeated after each rotation of pig pairs, see section “2.7 After testing” below.

### 2.3. The habituation/dishabituation paradigm

The test was adapted from Rørvang et al. (2017) in combination with Mendl et al. (2002), based on the habituation/dishabituation paradigm that repetitive presentation of the same odor will reduce interest (habituation to the odor), whereas subsequent exposure to a different odor will increase interest (dishabituation to the odor), indicating that pigs are able to detect and distinguish between the two presented odors (Sundberg et al., 1982). Animals tested in the habituation/dishabituation test are usually tested individually, but to reduce the risk of stress associated with social isolation, pigs in the current experiment were tested in littermate pairs with both animals (one male and one female) constituting the experimental unit (i.e., 192 pigs/2 = 96 pig pairs tested). In the test pen, each odor was tested alongside an odorless control (demineralized water), with odor presented in one and control in the other insertion point. The insertion points used for odors and controls (either left or right) were the same throughout one test day but were alternated the following test day (i.e., test day 1: odor left, control right; test day 2: odor right, control left and so forth). The left/right placement was mirrored between test pens, both across the central aisle, as well as between neighboring test pens. This placement limited potential odor cueing to the left/right between adjacent pens whilst balancing for potential laterality of the pigs.

### 2.4. Selection of odors

In this experiment, 12 fragrance oils of complex odors (i.e., odors composed of several different odorant molecules) approved for human use by The International Fragrance Association [IFRA] (2022), were used. The experimental odors of 8 essential oils and 4 synthetic perfume oils are listed in Table 1. The odorants in essential oils are derived from 100% natural sources, whereas the synthetic perfume oils used in this experiment were a blend of both natural and nature-identical odorant molecules. Selection of odors for testing was based on: (1) the limited existing knowledge of odor interest in pigs [e.g., (Van de Weerd et al., 2003; Oostindjer et al., 2011; Nowicki et al., 2015)], (2) odors being of a non-social origin, (3) current knowledge about olfactory capacities in other animal species [e.g., (van den Berg et al., 2016; Rørvang et al., 2017)], (4) the chemical information available for each compound, and (5) the avoidance of substances that could potentially affect meat quality (Deslandes et al., 2001) for full details about odor selection see Supplementary material). A person unrelated to the experiment was exposed to the odors in a “pilot sniffing test” to ensure that the human nose perceived the selected odors as being different. The 12 odors were then grouped in threes, based on their origin (herb, spice, from a tree, root, fruit, flower, or seed). This grouping was done to limit the risk of sniffing fatigue and/or loss of motivation to explore the odors, and thus each pig pair was tested on only three of the 12 odors (see Table 2 and section “2.6 Testing” below).

**TABLE 1:** List of the 12 odors used in the experiment divided into type of odor (essential oil or synthetic perfume) with Latin name, manufacturer, and batch number listed.

	Odor (Latin name)	Origin: manufacturer, city, country, batch number (plant part)
**Essential oil**		
1	Blood orange^1^ *(Citrus sinensis)*	Urtegaarden, Allingåbro, Denmark Batch number: 0320652065 (peel)
2	Aniseed *(Pimpinella anisum)*	Fischer Pure Nature, Fredensborg, Denmark Batch number: 09210021003-501 (seed)
3	Cedarwood *(Cedrus atlantica)*	Fischer Pure Nature, Fredensborg, Denmark Batch number: 09210061008404 (plant body/wood)
4	Ginger *(Zingiber officinale)*	Urtegaarden, Allingåbro, Denmark Batch number: 0120652200 (root)
5	Cinnamon bark *(Cinnamomum aromaticum)*	Urtegaarden, Allingåbro, Denmark Batch number: 0920652260 (wood/bark)
6	Lavender *(Lavendula angustifolia)*	Fischer Pure Nature, Fredensborg, Denmark Batch number: 1021271340 (flower)
7	Pine *(Pinus ssp. Pinaceae)*	Fischer Pure Nature, Fredensborg, Denmark Batch number: 1021A-14417 (needles)
8	Thyme *(Thymus vulgaris)*	Fischer Pure Nature, Fredensborg, Denmark Batch number: 08218007-46-3 (leaves)
**Synthetic perfume**		
9	Apple	Urtegaarden, Allingåbro, Denmark Batch number: 0820451389 (not specified)
10	Musk	Urtegaarden, Allingåbro, Denmark Batch number: 0920451230 (synthetic)
11	Vanilla^1^	Fischer Pure Nature, Fredensborg, Denmark Batch number: 092123993 (not specified)
12	Jasmine	Fischer Pure Nature, Fredensborg, Denmark Batch number: 092123939 (flower)

^1^Previously tested by Nowicki et al. (2015).

For detailed information on content listed in the compound safety data sheet for each odor, please see Supplementary Table 2.

**TABLE 2:** All odor presentation orders, i.e., odor sequences (A–Y) used.

Odor sequence	Odor no. 1	Odor no. 2	Odor no. 3	N_pig_ _pairs_
A	Vanilla	Aniseed	Blood orange	4
B	Musk	Apple	Cinnamon bark	4
C	Ginger	Pine	Jasmine	4
D	Cedarwood	Thyme	Lavender	4
E	Aniseed	Blood orange	Vanilla	4
F	Apple	Cinnamon bark	Musk	4
G	Pine	Jasmine	Ginger	4
H	Thyme	Lavender	Cedarwood	4
I	Blood orange	Aniseed	Vanilla	4
J	Cinnamon bark	Apple	Musk	4
K	Jasmine	Pine	Ginger	4
L	Lavender	Thyme	Cedarwood	4
M	Aniseed	Vanilla	Blood orange	4
N	Apple	Musk	Cinnamon bark	4
O	Pine	Ginger	Jasmine	4
P	Thyme	Cedarwood	Lavender	4
Q	Vanilla	Blood orange	Aniseed	4
R	Musk	Cinnamon bark	Apple	4
S	Ginger	Jasmine	Pine	4
T	Cedarwood	Lavender	Thyme	4
U	Blood orange	Vanilla	Aniseed	4
V	Cinnamon bark	Musk	Apple	4
X	Jasmine	Ginger	Pine	4
Y	Lavender	Cedarwood	Thyme	4

Each odor sequence was simultaneously tested on four pig pairs (two pig pairs each from two different litters). This was done to ensure that only one odor sequence was tested at a time, in order to limit odor contamination and ensure that no pigs had any prior exposure to the odors on which they were being tested (i.e., preserving novelty). Each odor sequence was tested simultaneously on four pig pairs, each odor was tested on 24 pig pairs (i.e., 96 pig pairs tested in total), and only one odor sequence was tested per day.

### 2.5. Preparation of odor samples

Prior to experimental start, all equipment was placed in the experimental building for approx. 2 months to allow potential odors from the new materials to dissipate. For each of the four test pens, four boxes were prepared before the testing commenced: one control and three different odor samples (16 boxes in total: four controls and 12 odor samples). This ensured that all four pens could be tested simultaneously and that all pig pairs were exposed to the same odors at the same time, thereby preventing any potential cross-contamination of odors.

Fresh odor samples were prepared on each test day in a separate, designated preparation room (for full details of the entire preparation procedure see Supplementary Information 1). An odor sample consisted of one piece of unbleached (i.e., light brown) filter paper placed in the odor box (dimensions (L × W × H): 21 × 17 × 15 cm, 3L, phthalate-free plastic and approved for human food). Six drops of the specific odor (or demineralized water for the control) were then added to the filter paper, with three drops on each end of the filter paper. The filter paper absorbed the oil/water and there were no visible (coloring) differences between the samples. All odors dissipated from the filter paper as all experimenters could easily detect the specific smell upon adding the odor oil, and when opening the boxes before each test. The odor box was sealed with a plastic lid to prevent the odors from dissipating and to minimize the risk of odor contamination. Control boxes were never used for odor presentations. When all four odor boxes had been prepared and sealed, they were transferred to a larger plastic container which was also sealed with a lid to further reduce the risk of odor contamination. When all odor/control boxes had been prepared, they were moved to the experimental building. The choice of using plastic (polypropylene) containers for the odors, was made for practical reasons, as glass containers would create an unnecessary risk of having broken glass near the animals.

### 2.6. Testing

A balanced odor presentation order (i.e., odor sequence) was defined before testing for all the groupings of three odors, to ensure that within a group of three odors (*n* = 4, Table 2) all possible combinations of the three were tested. This resulted in 24 different odor sequences (A–Y, Table 2), with each odor being included in six sequences (twice as 1st, 2nd, and 3rd odor, respectively), and each sequence tested on four pig pairs (i.e., each odor tested 24 times). Only one odor sequence was tested per day in the experimental building. Due to varying group/litter sizes, this resulted in a total of 50 litters being included and tested on 25 test days in total distributed over 5.5 months.

On each test day, the same odor sequence (and thereby also the same three odors) was assigned to all four pens/pig pairs by an experimenter not taking part in the actual tests, and before the experimenters arrived at the research center. This allocation of odors was done to eliminate the risk of companion pigs in non-test pens being exposed to the odors they would be tested on, prior to being tested themselves. The tests were performed during two designated time slots: one in the morning (9–11:00 h), and one in the afternoon (14–16:00 h), giving pigs the possibility to eat before each slot to limit the risk of any hunger effects. All tests were performed by two, out of the total three, participating experimenters in the study. All three experimenters received prior training in performing the tests and did not use any perfumed products for at least 48 h prior to the tests. On a test day, one experimenter performed the odor presentations and removals for test pens 1 and 2, while the other experimenter performed the same procedures for test pens 3 and 4.

**FIGURE 2:**
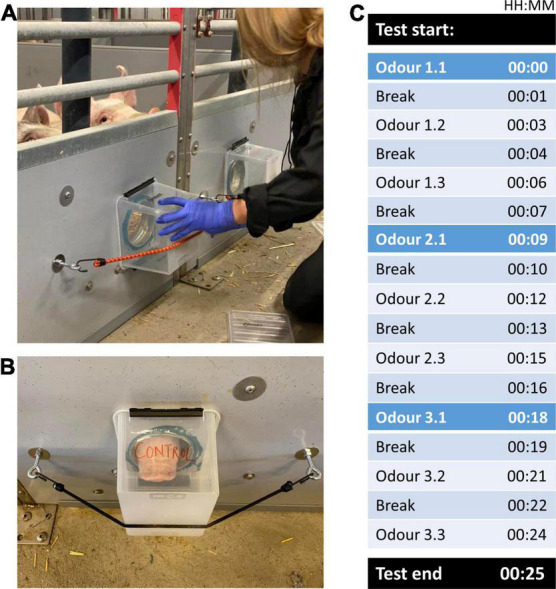
**(A)** The odor box being fitted to an odor insertion point, covering the entire hole in the pen wall; **(B)** the odor box was held in place by an elastic strap hooked into bolted-in metal loops on either side of the insertion point; **(C)** a schematic presentation of the testing protocol, with black rows illustrating test start and end, and each odor presentation (1 min duration) marked from 1.1 (1st odor presented for the first time) through to 3.3 (3rd odor presented for the third time), and inter-trials breaks (each 2 min duration).

Each of the three odors was presented three times in a row (giving a total of 9 presentations), where each odor presentation (trial) lasted for 1 min followed by a 2 min inter-trial break (Figures 2A–C). The timer for the 1st presentation of each odor was started once at least one of the two pigs in the experimental unit approached the insertion points, and thus was within a snout length (∼8 cm) of either odor or control. If pigs did not approach voluntarily, the experimenters tapped and/or scratched the boxes lightly to encourage the pigs to investigate. This was done to ensure that (a) pigs noted the odors and (b) any subsequent display of uninterest (no approach, no sniffing) in the odors was not unawareness misinterpreted. Both boxes were tapped/scratched simultaneously to avoid unintentional cueing to one specific side. The odor/control boxes remained fastened during the 1 min odor presentation (Figure 2B), before being removed and sealed with the lid for the 2 min inter-trial break (Figure 2C), during which the insertion points were left uncovered. After the 1st presentation of an odor, the 2nd and 3rd presentations of the odor commenced after the inter-trial breaks, regardless of whether pigs approached the boxes. After the last (3rd) presentation of an odor, the odor boxes were placed back in their container which was subsequently sealed with the lid. Following the 2 min inter-trial break, the next set of odor boxes was taken out of their container and presented to the pigs in the same way, with the same criterion for approach during the 1st presentation of the 2nd as well as 3rd odor (Figure 2C). As each experimenter handled two test pens at a time, each experimenter 1st started the testing in one pen before turning across the aisle to start the second pen. Except for when fitting and removing the boxes from the odor insertion points, the experimenters remained seated and quiet throughout the testing. The experimenters timed presentations and breaks for each pen by use of two stopwatches (one per test pen) of different models and colors for easy differentiation between the watches associated with each pen.

### 2.7. After testing

**FIGURE 3:**
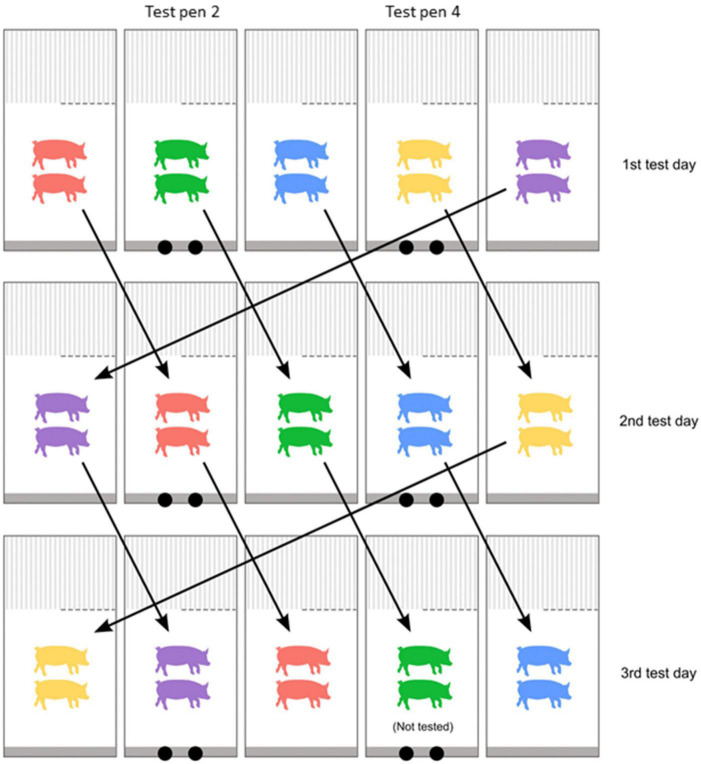
Pig rotation scheme adapted from Gadri (2022). Each square represents one of the five pens on either side of the central aisle of the experimental building, with test pens marked with two black circles representing the insertion points. Each color represents an individual pig pair. The arrows illustrate how all pairs were rotated after one full test day – the new placement of pairs on the subsequent 2nd test day. This rotation was repeated after each test day to ensure all pairs eventually were placed in a test pen on one of the days, until all pairs had been tested. The procedure was the same for both rows of test pens (Figure 1).

After the simultaneous testing of the four pig pairs, the experimenters moved all odor and control boxes from the experimental building back to the prep room. All odor boxes were cleaned with warm water and wiped dry with paper towels before being sealed with the lid. The insertion points were also cleaned with water and odorless soap inside and outside the pig pen. After each test day, all pig pairs were rotated, to occupy a new pen in the experimental building. This procedure was done following a pig rotation scheme (Figure 3). The pig rotation scheme ensured that all individual pig pairs were present in the test pens on separate days, and thus could be tested on one of the test days. No pig pairs were thus tested twice. The pig rotation scheme further limited the risk of aggression when the pig pairs were reunited in their home pens with their litter mates after the experiment, by allowing partial contact through the bars dividing the slatted areas of the pens (Gadri, 2022). After one rotation, the new pig pairs inside the test pens were allowed the same 24–29 h to acclimatize to the pens and the insertion points before a new round of testing was initiated. The testing and rotation procedures were repeated until all pairs, within the same two litters placed in the experimental building at the same time, had been tested. This resulted in the pigs spending at least 3 days and maximum 4 days inside the experimental building (depending on litter size).

### 2.8. Behavior

**FIGURE 4:**
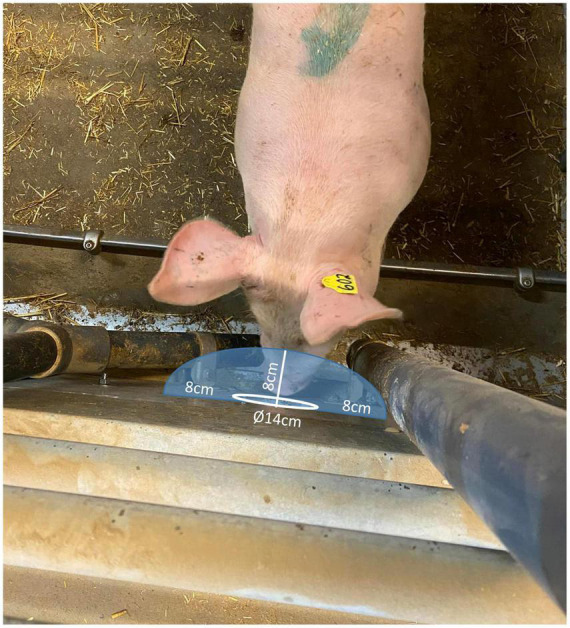
Illustration of the distance (8 cm from the rim or center of the hole indicated by the blue area) within which the snout of the pig had to be for the pig to be recorded as “sniffing the odor”.

All tests were video recorded (GoPro Hero 9), with one camera per pen, recording a top view of the front pen wall, central aisle and front half of the pig pen (covering the solid floor area, but not the slatted). From the video recordings, the behavior of the pigs was later extracted. All behavior described below was recorded separately for both insertion points (i.e., for the odor and the control insertion point separately), by an experienced observer who was blind to the specific odor being tested but not to the placement of odor/control to the left/right as experimenters always fitted the control box first (i.e., before fitting the odor box). During each odor presentation, the total sniffing behavior of each pig (i.e., sniffing duration summed for the pig pair per odor presentation) was recorded using continuous sampling of each 1 min odor presentation (Martin et al., 2021) and the combined duration was used in the analyses. “Sniffing behavior” was defined as: a pig was in close proximity to the insertion point (i.e., less than the length of a pig snout away from the insertion point, approx. 8 cm; Figure 4) or in direct contact with the insertion point (Figure 4). Habituation was defined as a significant decrease in sniffing of the odor between at least two of the three presentations of the same odor, and dishabituation was defined as a reinstatement of sniffing (significant increase in sniffing duration) when a new odor sample was presented. Definitions of all other behaviors are described in Table 3. Feeding-related behavior and agonistic behavior as well as no approach (Table 3) were continuously recorded using behavior sampling (Martin et al., 2021). Feeding-related behavior was recorded as a collective duration for both pigs during each odor presentation (as for sniffing). Agonistic behavior was also collectively recorded for both pigs during each presentation as duration per odor presentation.

**TABLE 3:** Ethogram of all recorded behaviors per trial.

Behavior category	Behavior	Description
Feeding related	Licking	Pig in direct contact with the insertion point, repeatedly opening and closing the mouth with tongue protruding.
	Biting	Same as licking, but with repeated biting (open mouth) of the edge of the insertion point, or fixtures around the point.
	Rooting	Pig in direct contact with the insertion point, while forcefully and repeatedly pushing with the snout the fixtures around the insertion point or the inside of the insertion point.
Agonistic	Displacement	One pig successfully displaces the other pig from the insertion point, and the other pig moves at least three steps back/away. Might include pushing and/or biting of the other pig prior to the successful displacement.
	Pushing	(Unsuccessful displacement) While one pig is sniffing the insertion point, the other leans toward the other pig or pushes with their head, snout or body. The receiving pig either leans against the weight or remains unmoved.
	Biting	While one pig is sniffing the insertion point, the other directs a bite with open mouth at the receiving pig’s head or body.
Other	No approach	The pig does not approach any of the insertion points (does not meet the sniffing criterion), or is out of sight of the camera lens i.e., far away from the insertion points.

### 2.9. Statistical analyses

All statistical analyses were performed in the online software R [version 4.1.0.; (R Core Team, 2018)], using the interface R-studio [version 2022.12.0 + 353; (RStudio Team, 2022)]. We used 5% as the significance level, and 10% as the level for tendencies.

As all pigs were tested in pairs, the experimental pig pair was the experimental unit. All analyses were thus done at pig pair level, where all pairs included one male and one female and investigations of potential effects of sex were thus not possible. The data consisted of nine repeated measures for each experimental pig pair; three presentations per odor, of a total of three odors per pair (Table 2). The total data set thus comprised 828 odor presentations. Out of these 828, 50 presentations (6%) were lost due to camera error, leaving 778 odor presentations for analysis.

To test for overall effects of side (left vs. right hole) and presence of an odor vs. control on sniffing duration, sniffing durations were compared (left vs. right hole and odor vs. control hole) in paired (dependent) *t*-tests across all odors. This was done prior to modeling to check for potential side bias (left vs. right bias) of the pigs and to check if the control was investigated less than the odor side.

#### 2.9.1. Habituation/dishabituation

Sniffing duration data for both the odor side and the control side were right-skewed due to a large number of zeros and was thus either log-transformed before being analyzed or analyzed using methods appropriate for non-normally distributed data. To investigate if habituation occurred over the three repeated presentations of the same odor, a Linear mixed-effect model (LMM) for normal data was fitted to the log-transformed data from each specific odor (i.e., in separate models per odor), using R-package lme4 (Bates et al., 2015). The 12 full LMM models all included fixed effect of trial (categorical variable with three levels: 1, 2, 3), breed (categorical variable with three levels), average pig pair bodyweight, and a random effect of pig pair ID (1–92) to account for repeated measures on each pig pair (i.e., three odor presentations of the same odor per pig pair). Stepwise reduction was applied in the model fitting stages (with *P* > 0.1 as the threshold), with breed as well as body weight excluded from all final models. Final models thus included the fixed effect of trial, and the random effect of pig pair ID. Residuals from each model were evaluated in QQ-plots. Statistical significance was evaluated using a linear regression ANOVA.

To investigate if dishabituation occurred between 3rd odor presentation of one odor and the 1st odor presentation of another odor, durations for these trials were analyzed in a separate data set. These data contained 2 odor comparisons per pig pair, and eight repetitions of each odor comparison (i.e., *n* = 8 for each 3rd and 1st comparison). Data were log-transformed and a LMM was fitted including fixed effects of trial (categorical variable with two levels: 3rd and 1st presentation) nested in “odor comparison number” [categorical variable with two levels: 1st (odor 1 and odor 2) or 2nd (odor 2 and odor 3) comparison] and odor. The model further included the random effect of pig pair ID (1–92) to account for repeated measures on each pig pair (i.e., two comparisons per pig pair). Stepwise reduction was applied in the model fitting stages (with *P* > 0.1 as the threshold), with breed as well as body weight and odor removed from the model. Residuals of the final model was evaluated in a QQ-plot, and statistical significance was investigated in an ANOVA. Pairwise comparisons of the fixed effect of trial nested in comparison number from the model were performed using contrasts in R-package emmeans (Lenth, 2022). Pairwise comparison was done to investigate if a significant increase in sniffing between 3rd and 1st presentation occurred in both 1st and 2nd odor comparisons.

#### 2.9.2. Control side sniffing and sniffing over time

Investigation of sniffing duration of the control side was done in the same manner as for the odor side but using the full data from all pigs and odors (*n* = 778 control odor presentations). Data were log-transformed and the full model (LMM) included the same fixed and random effects. Stepwise reduction was applied in the model fitting stages (with *P* > 0.1 as threshold), with breed as well as bodyweight removed in all models. Final models thus included the fixed effect of trial, and the random effect of pig pair ID. Residuals from the final model were evaluated in QQ-plots. Overall, statistical significance was evaluated using a linear regression ANOVA. Pairwise comparisons of the fixed effect of trial from the model were performed using contrasts in R-package emmeans (Lenth, 2022). This pairwise comparison was done to investigate if sniffing of the control side increased or decreased between successive trials (1–2, 1–3, and 2–3).

To investigate if sniffing duration changed over time regardless of odor, a LMM was fitted to the log-transformed data of sniffing duration of the odor side. This model included fixed effects of trial (1, 2, 3), odor number (1st, 2nd, and 3rd odor), breed, average pig pair bodyweight, and random effect of pig pair ID to account for repeated measures on each pig pair (i.e., nine odor presentations per pig pair). Once again, stepwise reduction was done (with *P* > 0.1 as threshold), with breed and bodyweight removed from the final model, and residuals were checked in QQ-plots. Overall statistical significance in the final model was evaluated using a linear regression ANOVA, and pairwise comparisons of the fixed effect of odor number from the model were performed using contrasts in R-package emmeans (Lenth, 2022). The pairwise comparison investigated if sniffing of the odor side increased or decreased over time/odor presentations (1st vs. 2nd, 1st vs. 3rd, and 2nd vs. 3rd odor).

#### 2.9.3. Interest in odors

To investigate if any odor elicited more/longer sniffing than others, sniffing durations were summed for each odor (total of trials 1, 2, and 3) and analyzed using a LMM of all odors collectively. These data were normally distributed, hence log-transformation was not needed. The full model included odor (categorical variable with twelve levels) and odor type (categorical variable with two levels: essential oil, synthetic perfume) as a fixed effects and a random effect of pig pair ID (1–92) to account for repeated measures on each pig pair and the fact that each odor was not tested on all pairs. Residuals were checked in a QQ-plot. Significance of the fixed effects of odor and odor type was evaluated using ANOVA.

#### 2.9.4. Behavior

The duration of feeding-related and agonistic behavior categories were summed, respectively, to form “total feeding-related” and “total agonistic behavior” before being analyzed due to the relatively low occurrence of each specific behavior category. When comparing the durations of each behavior (feeding-related and agonistic behavior separately) while exploring the odor vs. the control samples, data were compared using paired *t*-tests. Durations of each behavior were additionally analyzed in two separate LMM’s and of all odors collectively. The full models included odor (categorical variable with twelve levels), the interaction of odor and odor group, breed (categorical variable with four levels), average pig pair bodyweight as a fixed effect and a random effect of pig pair ID (1–92) to account for repeated measures on each pig pair. Stepwise reduction showed no effect of breed or body weight (*P* > 0.1) and both fixed effects were thus removed from the final models. Although the interaction between odor and odor group was insignificant, this fixed effect was kept to control for the grouping of odors. Significance of the fixed effect of odor was evaluated using ANOVA, and pairwise comparisons of the fixed effect of odor were performed using contrasts in R-package emmeans (Lenth, 2022).

## 3. Results

There was no overall effect of side (left vs. right insertion point) on sniffing duration (Paired *t*-test: *t* = 0.5, df = 773, *P* = 0.6), but there was an overall effect of odor vs. control (Paired *t*-test: *t* = 9.5, df = 773, *P* < 0.001), with pigs sniffing the odor side significantly longer than the control side.

### 3.1. Habituation/dishabituation

**FIGURE 5:**
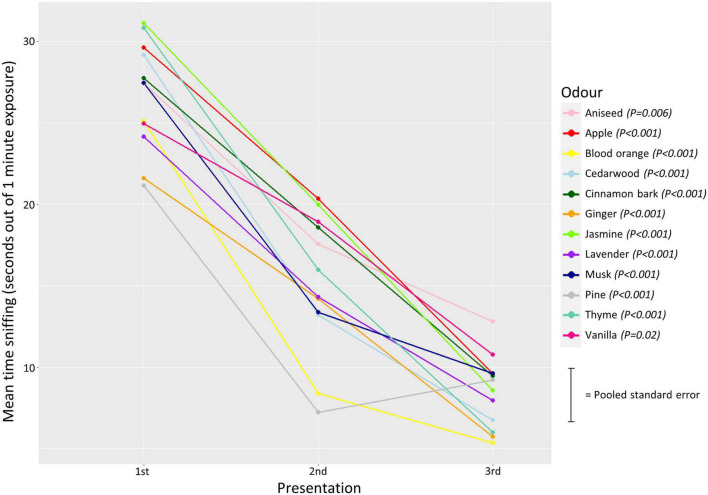
Mean sniffing duration in 1st, 2nd, and 3rd presentation of each odor represented by different colors. The lines connecting the means indicate a reduction in sniffing duration over each presentation. Error bars are excluded to allow easy identification of each odor (i.e., color), and each point, but a pooled standard error is indicated on the right. *P*-values from the linear regression analysis are given next to the odor.

The analyses showed that sniffing duration significantly reduced over repeated presentations of the same odor, for all odors included (Supplementary Table 2), hence habituation to the odors occurred in all cases (Figure 5).

**FIGURE 6:**
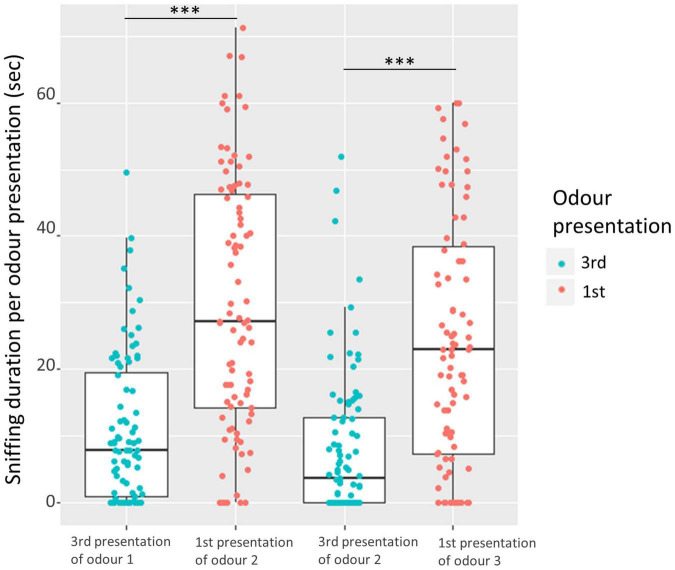
Comparison of sniffing duration from 3rd presentation of same odor to 1st presentation of a new odor (two comparisons), with each point representing each pig pair (3rd = blue, 1st = red). The bars represent the 25; 75% quartiles, the horizontal line within the bar represents the median, and the vertical lines illustrate the range. Asterisks illustrate the significance level of each comparison from the LLM (contrast odor 1 and 2, and contrast odor 2 and 3). Significance level marked as: ^***^(*P* < 0.001).

Dishabituation, i.e., significant reinstatement of sniffing from 3rd presentation of same odor to 1st presentation of a new odor also occurred (trial nested in comparison: χ^2^ = 177.7, df = 3, *P* < 0.001), both in the 1st odor comparison [LMM (contrast odors 1 and 2): estimate ± se = 18.9 ± 1.9 s, df = 255, *P* < 0.001] and the 2nd comparison [LMM (contrast odors 2 and 3): estimate ± se = 16.2 ± 1.9 s, df = 255, *P* < 0.001], Figure 6.

### 3.2. Control side sniffing and sniffing over time

Pigs’ sniffing of the control side was more frequent in 1st presentations than in 2nd and 3rd presentations (χ^2^ = 23.0, df = 2, *P* < 0.001).

The investigation of sniffing duration of the odor side showed that pigs sniffed the 1st odor longer than the 3rd odor [LMM (contrast 1st vs. 3rd odor): estimate ± se = 4.7 ± 1.2 s, df = 687, *t*-ratio = 4.0, *P* < 0.001], and there was a tendency for pigs to sniff the 2nd odor less than the 1st [LMM (contrast 1st vs. 2nd odor): estimate ± se = 2.6 ± 1.2 s, df = 684, *t*-ratio = 2.2, *P* = 0.07].

### 3.3. Interest in odors

**FIGURE 7:**
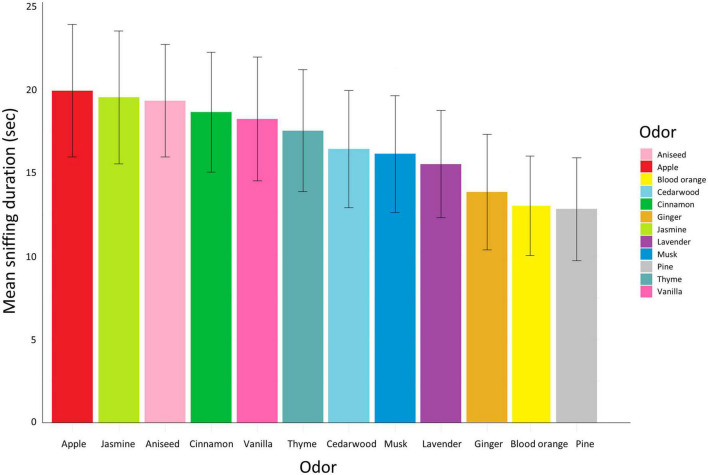
Mean sniffing duration per odor represented by different colors and ordered from highest to lowest mean duration. The error bars represent the standard error.

The mean (± se) duration of sniffing of each odor per pig pair is illustrated in Figure 7. Specific odor and odor type had no significant effect on sniffing duration (odor: χ^2^ = 11.3, df = 10, *P* = 0.3, odor type: χ^2^ = 0.1, df = 1, *P* = 0.7).

### 3.4. Behavior

Pigs were generally motivated to explore the odors. Only in 17% of the presentations (133 out of the 778 odor presentations) did the pigs not approach the odor or control (none of the pigs approached only the control or the odor side alone). No-approach behavior was not connected to any specific odor presentation number (1st, 2nd, or 3rd) nor specific odors, but pine had the numerically highest frequency (18 presentations) and aniseed the lowest (2 presentations).

**FIGURE 8:**
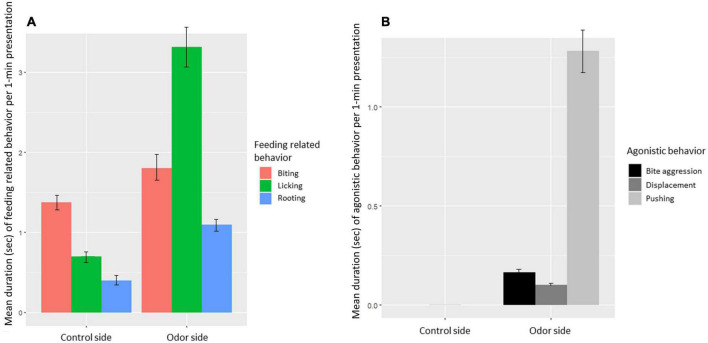
**(A)** Mean feeding-related behavior directed toward the odor or control (licking behavior, biting behavior, and rooting behavior) per odor presentation. **(B)** Mean agonistic behavior directed toward another pig (biting, displacements, and pushing behavior) per odor presentation at the control and the odor side.

Feeding-related behavior (Figure 8A) was expressed in 42% of the presentations (330 out of the 778 odor presentations). The most common feeding-related behavior was licking (Figure 8A). Agonistic behavior was expressed in 20% of the presentations (152 out of the 778 presentations; Figure 8B). Total feeding-related and total agonistic behavior were expressed significantly more for the odor side than for the control side (Paired *t*-test: feeding related: *t* = 8.1, df = 785, *P* < 0.001, agonistic behavior: *t* = 8.8, df = 785, *P* < 0.001).

Specific odors had a significant effect on expression of feeding-related behavior (χ^2^ = 26.7, df = 11, *P* = 0.005). A post-hoc contrasts analysis, however, showed that only jasmine and pine differed significantly, with jasmine eliciting significantly more feeding-related behavior than pine (LMM contrast: *t*-ratio = 4.1, df = 690, *P* = 0.002). None of the other odor comparisons differed significantly (LMM contrast all other odors: *P* > 0.1). There was a tendency for agonistic behavior to be affected by odor (χ^2^ = 19.3, df = 11.0, *P* = 0.06), but in the post-hoc contrasts analysis, none of the individual odors differed significantly (LMM contrast all odor comparisons: *P* > 0.05).

## 4. Discussion

The aims of this study were to identify which odors (among a sample of 12 odors of non-social origin) pigs were able to detect and distinguish between and record the behavior of the pigs when exposed to the odors, to evaluate odors as potential enrichment material. The results showed that pigs were able to detect and distinguish between odors in all four sets of three odors. As expected, sniffing duration decreased with repeated presentations of the same odor, indicating that the pigs habituated to all odors. When introduced to a new odor, a significant increase in sniffing duration occurred, indicative of dishabituation, i.e., the new odor was detected as being different from the previous one. The order by which the odors were presented also affected sniffing duration, with pigs displaying less sniffing as the test progressed (i.e., 2nd and 3rd odor). While pigs were generally motivated to explore all odors, and sniffed all odors longer than controls, no significant differences were found in sniffing duration between the different odors, or odor types. Lastly, pigs expressed both feeding-related behavior and agonistic behavior during the test, but mainly while exploring the odors as compared with the non-odor control. Jasmine elicited more feeding-related behavior than pine, but otherwise, none of the odors differed in their eliciting feeding-related or agonistic behavior.

### 4.1. Testing olfaction in pigs

The effect of odor number on pigs’ sniffing duration indicates that pigs were less motivated to investigate and sniff the odors being presented last. That pigs were more motivated to investigate the newest odor, demonstrates the importance of sustaining motivation (or novelty) for pigs to engage in a test paradigm such as the habituation/dishabituation test. This finding is in contrast to previous results using the same test paradigm and odors of non-social origin on horses (Rørvang et al., 2022), and cattle (Rørvang et al., 2017). In the cattle study, however, it was noted that the individuals quickly lost interest in investigating the odor-less control, implying a decrease in motivation to explore voluntarily, which the test paradigm relies on. In this design, we tried to prevent the latter effect from occurring by presenting the odors and the odorless control to the pigs at the same time. Although this method seemed to work well, the effect of odor number still indicates a shortcoming with the test. It is possible that the mere presence of the experimenters, equipment, and the test being performed, could have added novelty to the pigs’ environment in addition to the odors. This could be a contributing factor to explaining the effect of odor number, and the pigs’ willingness or motivation to engage for longer with the odors being presented first compared with the odors being presented last.

An alternative explanation might lie within the characteristics of the specific odors. The choice of plastic (polypropylene) for odor containers was made to make the results from the study applicable for on-farm conditions, but it is possible that the material could have affected the odors. Nevertheless, the containers remained the same throughout the experiment, making any potential effect the same across days and tests. Even if some odors reacted more with polypropylene than others, that particular odor would still have smelled the same to all pigs. More importantly, none of the odors used were diluted, in an attempt to ensure each odor being equally potent. It is, however, possible and even likely, that odors differed in how strong or potent each pig perceived them while exploring. Future studies should control for this aspect by using single-molecule odorants with known and tested dilution potencies, or testing gradient dilutions of individual odors to determine the detection points specifically for porcine olfaction (Søndergaard et al., 2010). A third explanation could be a form of sniffing fatigue (Adler and Finley, 1938), following repeated exposure to odors over a relatively short period of time (25 min). From human olfaction research, sniffing fatigue (or olfaction fatigue) is well-known, and the human nose will swiftly adapt when smelling the same odor after just a few seconds (for review on olfaction adaptation see: Cometto-Muñiz and Cain, 1995). If humans are to re-detect an odor, sniffing behavior needs to be interrupted, which means that, in our case, pigs with longer sniffing bouts might have temporarily lost the ability to smell the odor as a result, whereas pigs with a high number of sniffing interruptions may have continued to detect the odors.

Lastly, living in a barren environment with relatively low olfactory variation, such as a pig barn, and being a macrosmatic (highly developed olfactory organs and smell-dependent) animal, exposure to potent odors may have been a very strong stimulus for the pigs. It may be that the pigs were over-stimulated after investigating the 1st odor sample, resulting in less motivation (or olfactory ability) to engage with the subsequent odors, regardless of new odors being introduced. This could be remedied, at least in part, by expanding our knowledge on the thresholds for detection of the odors used in future studies.

Although these highlighted challenges persist, the habituation/dishabituation test paradigm for testing olfactory abilities of pigs is still a practical test for use in commercial production settings. The paradigm might not be suited for testing three or more odors, and may be more reliable if pigs were presented with just two consecutive odors to ensure motivation is sustained. The test does not require training and adaptation of the pigs, nor the use of rewards such as food/treats. It is therefore a good proxy for pigs’ natural ability and motivation to investigate and discriminate odors. Further adaptations of the test for pigs could include presentation of the odor samples at floor level. Pigs naturally sniff and root the ground, making odor presentation at ground level more biologically relevant. In this study, we presented the odors at the lowest position possible, whereas Mendl et al. (2002) presented their odor samples above the pigs’ heads. However, future studies could allow the pigs to investigate odors at ground level, possibly with an option to physically manipulate the sample by rooting.

### 4.2. Pigs’ interest in the odors and potential for use as enrichment

#### 4.2.1. Factors affecting pigs’ interest in odors

In this study, none of the odors elicited significantly more sniffing than others, although numerical differences in sniffing duration among the twelve odors were found. The type of odor (essential oils and synthetic perfumes) also did not affect the amount of sniffing elicited. All odors were approached and investigated, and absence of approach behavior was rare, indicating that pigs were motivated to explore the odors. It must be noted that each pig pair was tested on only three odors (to limit sniffing fatigue and sustain motivation to investigate the odors). Each group of three odors was, however, tested on the same number of pigs and the sample size (*n* = 24) was chosen based on previous studies to have sufficient power within each odor sub-set.

Another possible explanation for the absence of significant effects of odor or odor type on sniffing duration is individual variation. Individual perceptions of some odors being more or less pleasant may have affected the results. From research on human olfaction, a number of individual factors have been found to affect how an odor is perceived. (1) age: olfactory function and sensitivity declines with age, especially humans over the age of 80 have olfactory deficits, (2) sex: females outperform males in olfactory sensitivity and acuity, (3) internal state: hunger or satiety can alter sensitivity, (4) chemical pollution: various chemical compounds can affect or even damage olfaction, and (5) prior experience: prior exposure can affect the perception of subsequent exposure to the same odor (or to a different odor), for instance, an unpleasant odor may become less unpleasant with repeated exposure [for review see: (Doty, 2015)]. Research on these effects in non-human animals is sparse, but from working dogs, age has also been shown to affect olfaction (Jenkins et al., 2018), whereas results from horses are conflicting [no age effect: (Hothersall et al., 2010; Rørvang et al., 2022)]. In this study, age did not affect sniffing duration, but all pigs tested were relatively young (32–110 days old), and age effects are unlikely to occur until much later in life. Sex differences, similar to that of humans, have also been shown in non-human mammals [mice: (Kass et al., 2017), chimpanzees: (Matsumoto-Oda et al., 2007)] but in the current study, the pair testing did not allow to test for sex differences. In terms of internal state, all pigs were tested in a social setting to minimize stress from being socially isolated, and all pigs were tested in two 2 h periods to limit the risk of a hunger/satiety effect. With regard to chemical pollution, such as ammonia and hydrogen sulfide (Koerkamp et al., 1998; Seedorf and Hartung, 1999; Donham, 2000), all tests were carried out in the same conditions, and the ventilation system was similar to that of a normal conventional pig barn. This did not eliminate potential chemical pollution but made the results applicable to commercial housing where pigs would be in a similar odor-scape. Lastly, in terms of novelty of the odors, we assumed that the odors used had not been encountered previously by the pigs, as the twelve odors were not present in the pig feed, bedding nor in any treatments used on farm (e.g., wound treatment cream or similar). It was therefore unlikely that the pigs had any prior association with the odors which could have affected the results. It would be highly relevant for future studies to investigate one or more of the above-mentioned factors to further our knowledge of what affects pig olfaction.

#### 4.2.2. Odors as a resource to pigs

Licking, biting and rooting behavior was observed when pigs explored the odors, with licking behavior being the most prevalent. All feeding-related behaviors were more frequently directed at the insertion point containing the odor than the control. This may simply reflect that the pigs spend more time at the odor insertion point, but it could also indicate that the odors, in addition to stimulating the olfactory system, activated the facial and glossopharyngeal nerve (taste innervation of the tongue), and the trigeminal nerve (Cometto-Muñiz and Cain, 1995). Pigs also have a well functioning vomeronasal organ [e.g., (Guiraudie et al., 2003)], and the pigs may have perceived the odors as being edible if pigs can associate an odor with a taste, which has been demonstrated in humans (Schifferstein et al., 2022) and in horses (Rørvang et al., 2022). Given the (assumed) novelty of the odors to the pigs, it may be that novel odors, even of non-social origin, are of innate interest to pigs. Future studies on how pigs perceive odors should include neurobiological measures of olfactory processing to elucidate which areas of the brain is involved when pigs explore odors.

Out of the 12 odors included, two differed significantly in the elicitation of feeding-related behaviors. Jasmine, a synthetic perfume, elicited significantly more feeding-related behavior than pine, an essential oil. Pine, in addition, also had the numerically highest number of no-approach incidents, and the lowest total mean sniffing duration. This could indicate that pine was the least interesting odor for the pigs. However, pine elicited a large amount of rubbing and rolling behavior in the pigs (Rørvang et al., in review)^1^ and these behaviors, although poorly understood, are thought to be indicative of pleasure or at least a positive valence (Gosling and McKay, 1990; Hepper and Wells, 2017). This would indicate that pine was not perceived as edible, but that it had other attractive and still unknown properties.

The study may not yield sufficient information to indicate which odors have the most potential as enrichment for pigs, but it does provide some evidence for odors being a valued resource to pigs. No-approach behavior was infrequent, and pigs were motivated to investigate the odors, at least during the 1st presentations. Both agonistic and feeding-related behaviors were almost exclusively observed close to the odor insertion point, compared to the odor-less control point. The expression of agonistic behaviors close to the odor indicates that pigs were willing to pay (make an effort) for access to the odors, and/or defend their position at the odor point. Together with the expression of feeding-related behavior, these results indicate that pigs perceived the odors as a resource, which was potentially edible.

## 5. Conclusion

The results of this study collectively point to odors of non-social origin evoking an immediate interest in pigs. There was no indication of any particular odor or odor type being of more interest to the pigs and generally, all odors elicited both sniffing, agonistic and feeding-related behavior. Future research should focus on investigating detection thresholds of pigs for different odorants, pigs’ motivation for odor exploration over longer periods of time, and the practicalities of incorporating odors as enrichment, as well as its subsequent effect on pigs’ behavior and welfare.

## Data availability statement

The raw data supporting the conclusions of this article is publicly available via: https://doi.org/10.5878/kc64-wd49, and further questions regarding the data may be directed to: MR, mariav.rorvang@slu.se.

## Ethics statement

This animal study was reviewed and approved by the Swedish Board of Agriculture’s Uppsala Ethics Committee on Animal Research (ethics approval number: Dnr. 5.2.18-02900/2020).

## Author contributions

MR: conceptualization, methodology, formal analysis, investigation, resources, data curation, writing—original draft, writing—review and editing, supervision, project administration, and funding acquisition. S-LS: conceptualization, writing—original draft, writing—review and editing, and funding acquisition. JS: methodology, investigation, writing—original draft, and writing—review and editing. RG and MG: investigation and writing—review and editing. AV and BN: conceptualization, methodology, writing—review and editing, and funding acquisition. AW: conceptualization, methodology, writing—original draft, writing—review and editing, supervision, and funding acquisition. All authors contributed to the article and approved the submitted version.

## Footnotes

1 Rørvang, M. V., Schild, S.-L. A., Wallenbeck, A., Stenfelt, J., Grut, R., Valros, A., et al. (in review). Rub ‘n roll – pigs express rubbing and rolling behaviour when exposed to odours. Ani. Behav.
