# Supplementary material for: Odor exploration behavior of the domestic pig (Sus scrofa) as indicator of enriching properties of odors
Source: Front Behav Neurosci. 2023 May 5;17:1173298. doi: 10.3389/fnbeh.2023.1173298 (PMC10196037; doi:10.3389/fnbeh.2023.1173298)
Supplement: Supplementary file 1 [file Table_1.DOCX]

**Supplementary information S1**

*Selection and preparation of odor samples*

Prior to experimental start, all equipment was placed in the experimental building for approx. 2 months to allow potential odors from the new materials to dissipate. For each test pen (n=4), four boxes were prepared before the testing commenced: one control and three different odor samples (16 boxes in total: 4 controls and 12 odor samples). This ensured that all four pens could be tested simultaneously and that all pig pairs were exposed to the same odors at the same time, thereby preventing any potential cross-contamination of odors. Fresh odor samples were prepared on each test day in a separate, designated preparation room (“the prep room”), positioned behind three doors 200m from the pigs, to prevent odor contamination of the experimental building. All preparations were done by an experienced experimenter wearing latex gloves and who had not used any perfumed products (e.g. soap, shampoo, perfume, etc.) for at least 48h prior to prepping. Odor samples were made by placing one piece (12cm x 4cm) of unbleached (i.e. light brown) filter paper (manufacturer: Axfood AB, 107 69 Stockholm, Sweden, FSC certified paper) in the specific odor box (dimensions (LxWxH): 21cm x 17cm x 15cm, 3L, model: Smart StoreTM Classic 3, manufacturer: Orthex Sweden AB, 362 21 Tingsryd, Sweden, phthalate free and approved for human food). The filter paper was attached to the bottom of the odor box using a small piece of duct tape. Six drops of the specific odor (or demineralized water for the control) were then added at room temperature (20◦C), with 3 drops on each end of the filter paper. The filter paper absorbed the oil/water and there were no visible (coloring) differences between the samples. The odor box was swiftly sealed with a plastic lid to prevent the odors from dissipating and to minimize the risk of odor contamination. Each odor box was designated for the presentation of one specific odor, and always stored with its lid on to avoid odor contamination between test days. Control boxes were never used for odor presentations.

When all four odor boxes had been prepared and sealed, they were transferred to a larger plastic container which was also sealed with a lid to further reduce the risk of odor contamination. The samples of the remaining two odors and the controls were prepared in the same way, and all boxes of the same odor were made and transferred to their larger container before preparing any new odor/control. Latex gloves were changed between the handling of each odor/control. When all odor/control boxes had been prepared, the four larger containers (3 odor, 1 control) were moved to the experimental building.

**Supplementary Table S2**

List of the 12 odours used in the experiment divided by type of odour oil (essential or synthetic perfume) with Latin name, manufacturer, batch number and content (compounds listed in the safety data sheet) listed.

|  | *Odour (Latin name)* | *Origin: manufacturer, city, country,*  *batch number (plant part)* | *Content (compounds declared in the safety data*  *Sheet as potential allergens)* |
| --- | --- | --- | --- |
|  | Essential oil: | |  |
| 1 | Blood orange^1^ *(Citrus sinensis)* | Urtegaarden, Allingåbro, Denmark  Batch number: 0320652065 (cold pressed from peel) | Limonene 95-100%  Linalool 1-5%  Citral ≤2.5%  Myrcene ≤2.5% |
| 2 | Aniseed *(Pimpinella Anisum L)* | Fischer Pure Nature, Fredensborg, Denmark  Batch number: 09210021003-501 (water vapour distillation from seed) | Linalool 1-10%  Limonene ≤0.2%  Eugenol ≤0.2% |
| 3 | Cedarwood *(Cedrus atlantica)* | Fischer Pure Nature, Fredensborg, Denmark  Batch number: 09210061008404 (distillation of wood and bark) | Farnesol ≤2.3% |
| 4 | Ginger *(Zingiber officinale)* | Urtegaarden, Allingåbro, Denmark  Batch number: 0120652200 (water vapour distillation of root) | Camphene 5-10%  Alpha Pinene 1-5%  Limonene 0-4%  Terpineol 0.1-1%  Linalool 0.1-1%  Beta Pinene 0.1-1%  Myrcene 0.1-1%  Citral 0.1-1%  Para cymene 0.1-1% |
| 5 | Cinnamon bark *(Cinnamomum aromaticum)* | Urtegaarden, Allingåbro, Denmark  Batch number: 0920652260 (water vapour distillation of bark) | Cinnamonum Zeylanicum bark oil 100% |
| 6 | Lavender *(Lavendula Angustifolia)* | Fischer Pure Nature, Fredensborg, Denmark  Batch number: 1021271340 (distillation from flowers) | Linalool ≤40%  Limonene ≤1.2%  Geraniol ≤1% |
| 7 | Pine *(Pinus ssp. Pinaceae)* | Fischer Pure Nature, Fredensborg, Denmark  Batch number: 1021A-14417 (distillation from wood) | Limonene ≤8%  Limonene ≤5%  Linalool ≤1.5%  Geraniol ≤0.2% |
| 8 | Thyme *(Thymus Vulgaris)* | Fischer Pure Nature, Fredensborg, Denmark  Batch number: 08218007-46-3 (distilled from leaves) | Linalool ≤8%  Benzylbenzoat ≤1%  Geraniol≤0.5% |
|  | Synthetic perfume: | |  |
| 9 | Apple | Urtegaarden, Allingåbro, Denmark  Batch number: 0820451389 (made from natural extracts approved by IFRA^2^, excl. muscxylenes) | Benzenethanol 5-10%  Dihydromyrcenol 5-10%  4-undecanolide 1-5%  2-propenylhexanoate 1-5%  Terpinylacetat 1-5%  Fenylaetylacetat 1-5%  2,4-dimety-3-cyclohexen-1-carboxaldehyde 0.1-1% |
| 10 | Musc | Urtegaarden, Allingåbro, Denmark  Batch number: 0920451230 (synthetic, made from natural extracts approved by IFRA^2^) | Bergamot oil 10-20%  Linalool 5-10%  Vanillin 5-10%  Benzenethanol 5-10%  Benzenepropanal,apha-methyl-4-(1-methyl)- 5-10%  Benzylalcohol 5-10%  Linalylacetat 5-10%  2H-1-Benzopyran-2-on 5-10%  Petitgrainolie 5-10%  Cedarwood oil 1-5%  Lavender oil 1-5%  Patchouli oil 1-5%  Muskatelsalvie oil 1-5%  6-Octen-1-Ol-, 3,7-dimethyl- 1-5%  Benzylacetat 1-5%  Duodecylaldehyd C-12 0.1-1%  Citrus eucalypthus oil 0.1-1% |
| 11 | Vanilla^1^ | Fischer Pure Nature, Fredensborg, Denmark  Batch number: 092123993 (made from compounds approved by IFRA^2^) | Benzyl alcohol 60-80% |
| 12 | Jasmine | Fischer Pure Nature, Fredensborg, Denmark  Batch number: 092123939 (made from natural compounds approved by IFRA^2^) | Benzyl acetat 20-40%  Linalool 10-20%  6-Octen-1-Ol, 3,7-dimethyl- 10-20%  Linalylacetat 10-20%  Benzenethanol 1-5%  Petitgrain oil 1-5%  Vanillin 1-5%  Fenylacetaldehyd 1-5%  Indol 0.1-1%  Citrus eucalyptus oil 0.1-1%  Benzyl benzoate 0.1-1%  Ylang ylang oil 0.1-1%  Orange oil 0.1-1%  4-methylanisole 0.1-1%  Bergamot oil 0.1-1%  Duodecylaldehyd 0.1-1% |
| ^1^ previously tested by Nowicki et al., 2015  ^2^ IFRA = International Fragrance Association Procedure Codex | | |  |
